# Supplementary material for: Complexome profiling of the Chlamydomonas psb28 mutant reveals TEF5 as an early PSII assembly factor
Source: Plant Cell. 2025 Mar 17;37(6):koaf055. doi: 10.1093/plcell/koaf055 (PMC12164590; doi:10.1093/plcell/koaf055)
Supplement: koaf055_Supplementary_Data [file koaf055_supplementary_data.zip › psb28_interactive.zip!/plots/intensity/qualityPlots/peptides/ACP1_Cre16.g673109.t1.1.html]

### Cre16.g673109.t1.1

Trivial name: ACP1  
SeqCoverage [median]: 14.10  
Mass: 13.67 kDa  
  
 

| Sequence | MissedCleavage | Mass [Da] | wtIntensity | muIntensity | Score |
| --- | --- | --- | --- | --- | --- |
| HFEKIDASK | 1 | 1073.55 | 2471.22 | 5685.02 | 170.85 |
| VSPAASFEK | 0 | 934.48 | 15731.26 | 25519.71 | 89.70 |
|  |  |  | 18202.5 | 31204.7 |  |
